# Grade-control outdoor turning flight of robo-pigeon with quantitative stimulus parameters

**DOI:** 10.3389/fnbot.2023.1143601

**Published:** 2023-04-17

**Authors:** Ke Fang, Hao Mei, Yezhong Tang, Wenbo Wang, Hao Wang, Zhouyi Wang, Zhendong Dai

**Affiliations:** ^1^Institute of Bio-Inspired Structure and Surface Engineering, College of Mechanical and Electrical Engineering, Nanjing University of Aeronautics and Astronautics, Nanjing, Jiangsu, China; ^2^Chengdu Institute of Biology, Chinese Academy of Sciences, Chengdu, Sichuan, China

**Keywords:** animal robots, robo-pigeon, electrical microstimulation, stimulation parameters, turning flight control

## Abstract

**Introduction:**

The robo-pigeon using homing pigeons as a motion carrier has great potential in search and rescue operations due to its superior weight-bearing capacity and sustained flight capabilities. However, before deploying such robo-pigeons, it is necessary to establish a safe, stable, and long-term effective neuro-electrical stimulation interface and quantify the motion responses to various stimuli.

**Methods:**

In this study, we investigated the effects of stimulation variables such as stimulation frequency (SF), stimulation duration (SD), and inter-stimulus interval (ISI) on the turning flight control of robo-pigeons outdoors, and evaluated the efficiency and accuracy of turning flight behavior accordingly.

**Results:**

The results showed that the turning angle can be significantly controlled by appropriately increasing SF and SD. Increasing ISI can significantly control the turning radius of robotic pigeons. The success rate of turning flight control decreases significantly when the stimulation parameters exceed SF > 100 Hz or SD > 5 s. Thus, the robo-pigeon's turning angle from 15 to 55° and turning radius from 25 to 135 m could be controlled in a graded manner by selecting varying stimulus variables.

**Discussion:**

These findings can be used to optimize the stimulation strategy of robo-pigeons to achieve precise control of their turning flight behavior outdoors. The results also suggest that robo-pigeons have potential for use in search and rescue operations where precise control of flight behavior is required.

## 1. Introduction

The new trend in the development of robotic technology is the integration of animals and machines into animal robots (also known as cyborgs and bio-robots) (Webb, 2000; Zhang et al., 2018; Gao et al., 2019; Romano et al., 2019), which utilize the animal's body as motion carriers, and are controlled or manipulated of animal robots' movements by modulating neural signals through the brain-computer interface (BCI) with stimulates the specific brain areas (Birbaumer, 2006; Lebedev and Nicolelis, 2006; Bozkurt et al., 2009). Such robots have a wide range of promising applications, with great value in areas such as exploring the structure and function of the brain, studying animal locomotor behavior and defending public safety (Wang et al., 2013; Latif et al., 2015; Bozkurt et al., 2016). Currently, animal robotics research covers the entire airspace of water, land and air, and has achieved the preliminary behavioral modulation of various animal robots [mammals (Talwar et al., 2002), fish (Kobayashi et al., 2009), insects (Kobayashi et al., 2009) and birds (Cai et al., 2015)]. For the flying animal robots, the wide range of activities allows for greater application space. As a kind of animal robot with super heavy load and sustainable flight ability, robo-pigeon has natural advantages in long-distance flight control (Wang et al., 2018). In previous studies on robo-pigeons, the *posterior amygdala* (PoA), a fear-receptive area in the pigeon brain, and the *dorsalis intermedius ventralis anterior* (DIVA), a somatic nociceptive area, have been successfully stimulated to control motor behavior such as take-off and turning (Yang et al., 2015). However, this prolonged negative stimulation can cause non-adaptive physiological responses in pigeons, which can affect the reliability of their motor behavioral control. Therefore, in recent studies, the *formatio reticularis medialis mesencephali* (FRM) of the midbrain motor area in pigeons has been used as the main nucleus regulating the turning motor behavior of robotic pigeons. These results show that the flight trajectory of pigeons can be significantly modulated by varying the levels of microstimulation parameters (amplitude, frequency and duty cycle); the flight behavior of robot-pigeons can be controlled in an outdoor environment using a back-mounted miniature wireless neurostimulator and the pre-programmed hierarchical stimulus algorithm (Wang et al., 2018; Zhao et al., 2019). Despite these advances, precise control of the flight behavior of robo-pigeons from a perspective of brain function and structure remains a challenge due to the insufficient research on the neural structure and information pathways of the animal carrier itself. Therefore, in order to achieve precise control of the turning flight behavior of robo-pigeons, it is necessary to quantify the relationship between electrical stimulation and motor response.

In the study of motor control in animal robots, electrical micro-stimulation is a highly responsive, less fatiguing and highly reproducible method of stimulation (Kobayashi et al., 2009; Wang et al., 2018). The first animal robot was achieved in a cockroach in which the locomotion along a straight line was performed using electrical stimulation in the tactile receptor area (Holzer and Shimoyama, 1997). A similar attempt in rat motor control, i.e., robo-rat, was accomplished by electrically stimulating the whisker receptor area of the brain (Talwar et al., 2002). In addition, recent studies have shown that establishing quantitative relationships between stimulus parameter inputs and motor responses using electrical stimulation enables precise control of directional motor behavior in cockroach robots (Erickson et al., 2015), and quantitative adjustment of electrical stimulation parameters in the ventral posteromedial nucleus (VPM) can accurately control the steering angle of a rat robot (Xu et al., 2016). These studies have shown that there are three key factors in accurately inducing and control of the motor behavior of animal robots using electrical stimulation methods: intracerebral stimulation sites, stimulation signal type, and stimulation parameter patterns (Zheng et al., 2011). Precisely locating the intracerebral stimulation sites would determine the accomplishment of animal robot development. The most commonly used type of stimulation signal is a constant current, cathode-leading, biphasic square waveform (Erickson et al., 2015; Xu et al., 2016; Kong et al., 2019; Zhao et al., 2019). This stimulation signal has been demonstrated as being the safest and most effective, not only for achieving charge balance by alternating the polarity of the pulse phases, thereby reducing tissue damage and preventing electrode polarization (Lilly et al., 1955; Merrill et al., 2005; Reilly and Diamant, 2011), but also to have a greater ability to evoke neural excitation at cortical depths (Lilly et al., 1955).

In general, the location of stimulation sites and the type of stimulation signal in the brain are fixed in the motor control of the robo-pigeon, so the selection of the appropriate electrical stimulation parameters is crucial in controlling the turning flight behavior of the robo-pigeon. At present, electrical micro-stimulation parameters have been shown to encode alternative sensory information that is converted to the corresponding motor output by the central nervous system, while some of them can cause non-negligibly tissue damage when the exceeding stimulations, e.g., strength and/or duration, were presented, which constricts largely their applications (Merrill et al., 2005; Bari et al., 2013). While in outdoor field tests, tiny modifications in electrical stimulation para meters often resulted in significant behavioral changes in outdoor flights due to interference with various external environmental factors. For this reason, it is necessary to understand the effect of each signal parameter on the turning flight behavior of the robo-pigeon in order to overcome these limitations and also ensure its safe and stable flight. As well, since the electrical stimulation signal is only a pre-set unit signal that mimics the action potential, it is difficult to be consistent with the current inherent neural information characteristics (Adrian, 1968; Tehovnik, 1996), and even for the same motor behavior, the neuronal electrical signals are not activated simultaneously, nor are they of a single frequency and pulse width (Tehovnik, 1996). Therefore, setting different stimulation parameter modes, and quantifying the relationship between stimulation parameter input and motor behavior output, is a necessary condition to achieve precise control of animal robots.

To this end, we speculate that there should be a quantitative relationship between input stimulus parameters and turning flight speed and turn curvature of the robo-pigeon under outdoor flight conditions, and that the turning angle and turning flight radius can be controlled hierarchically by quantitative input of stimulus parameters. To test our hypothesis, we applied three adjustable parameters, i.e., stimulus frequency, stimulus duration, and inter-stimulus interval, to explore the turning flight behavior of the robo-pigeons. This study aims to demonstrate that electrical stimulation of the midbrain FRM region of pigeons can provide a stable and efficient, precise and quantitative method for hierarchical control of the turning flight behavior of robo-pigeons, with eventual application in navigation systems.

## 2. Materials and methods

### 2.1. Animals

All homing pigeons (*Columba livia*) were bred and housed in a loft under a normal day/night light condition and were trained daily to fly around the loft twice a day. Water, grit and standard pigeon food mix were available *ad libitum*. They were trained to adapt to loading a dummy weight nut (~16 g, nearly the same size and weight as the onboard control module) on their backs by gluing Velcro straps. Right before the start of the experiment, 20 homing pigeons of unknown gender (age = 1–1.5 years) were selected to complete at least 15 solo flights from the release site to ensure that they all had homing experience. All pigeons were transported to the release site in Jiangjun Mountain of Nanjing (118.78784°E, 31.93707°N), ~13.5 km from the homing loft. By the end of this training phase, those pigeons that formed independent and stable homing routes, according to the GPS data, were candidates for robo-pigeons.

### 2.2. Surgery

Twelve subjects were chosen as robo-pigeons for brain surgery and their body weight was 430 ± 35 g at the time of surgery. Surgical procedures were similar to those described previously (Cai et al., 2015). Briefly, animals were anesthetized with a subcutaneous injection of 1.5% aqueous sodium pentobarbital (32 g/ml, IP) and measures were taken to minimize their distress.

**Figure 1:**
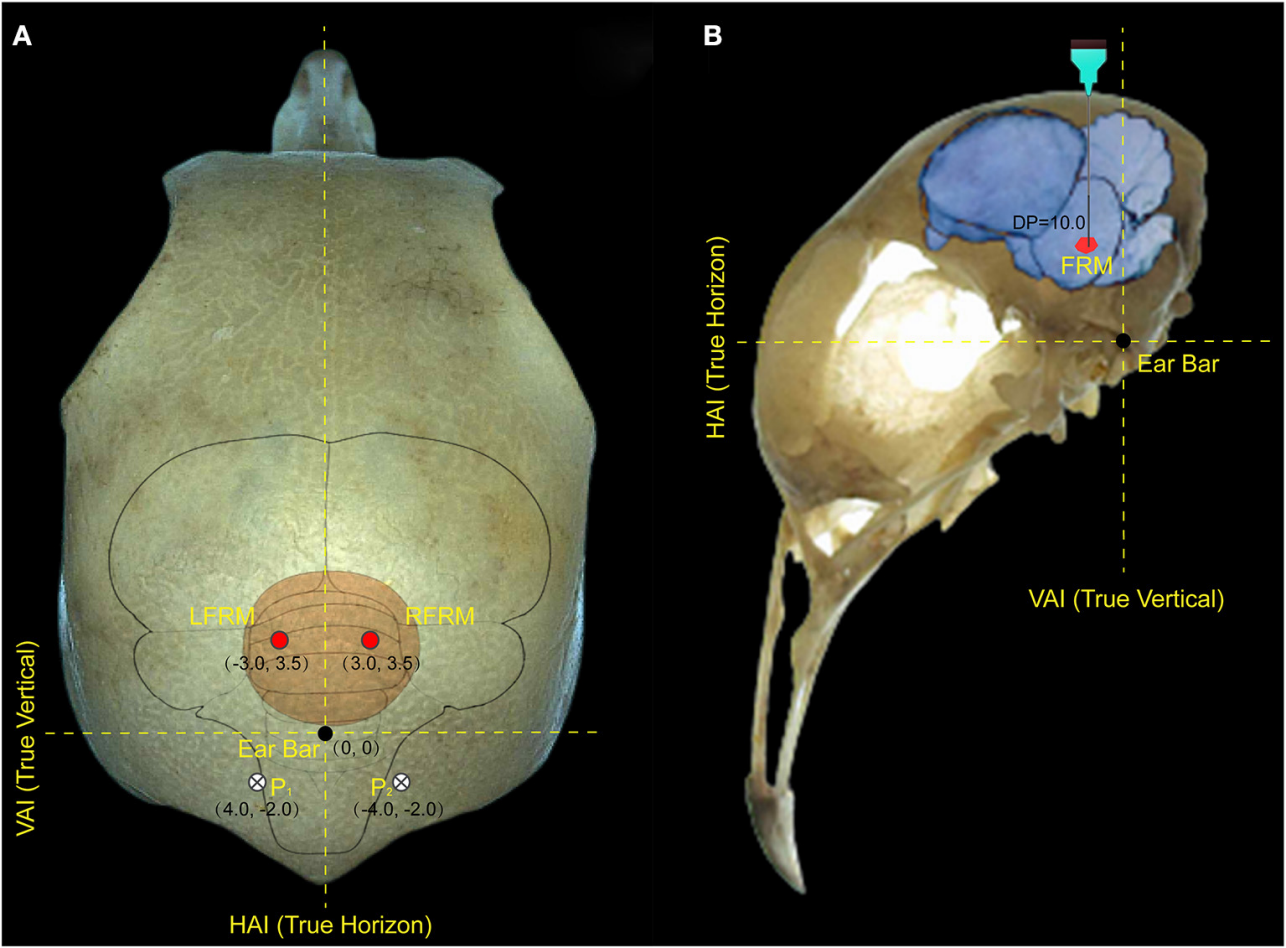
Locations of electrode implantation in the pigeon brain. **(A)** represents the implantation position of the electrode on the surface of the pigeon skull, and **(B)** represents the implantation depth of the electrode in the pigeon brain. The intersection of the true vertical lines and true horizon lines in the pigeon head denotes the center of the ear bar. LFRM, RFRM denotes the left and right sides of formatio reticularis medialis mesencephali, respectively; HAI and VAI represent the horizontal and vertical axes of the brain stereotaxic apparatus, respectively; while P1 and P2 denotes the reference electrode implanted above the biparietal suture lateral.

Anesthesia for surgery was confirmed if foot clipping did not induce withdrawal. After the anesthetic took effect, head feathers were shaved and 1% lidocaine (0.5–0.6 ml) was injected subcutaneously as a further local anesthetic. Anesthesia levels were assessed throughout the experiment and a supplementary dose (1/10 of the initial dosage, IP) was administered when a withdrawal response reappeared. Then, the pigeon was immobilized in a specially designed brain stereotaxic apparatus (Type 68027, RWD Life Science, Shenzhen China), and the location of the FRM area was selected as the target for microstimulation according to the pigeon brain atlas (Karten and Hodos, 1967). Each electrode lead was formvar-insulated nichrome wire (diameter 100 μm) with one end implanted into the left or right FRM area (AP: 3.5 mm, ML: 3.0 mm, DP: 10.0 mm) and the other end was tin-soldered to the electrical adapter. Two screws were implanted on the skull surface at P1 and P2 respectively, and a silver wire was wrapped around the screws as the reference pole (Figure 1). All electrode implantation sites were sealed with α-cyanoacrylate quick medical adhesive (EC) to seal the gap between the screw and the skull and then fixed with dental acrylic. The robo-pigeon was housed individually in an iron wire cage (59 × 26 × 52 cm) containing water and food for 6 days in recovery period before further experiments. After all experiments were completed, the subjects were injected with an overdose of pentobarbital sodium solution, and the brains were fixed by perfusion of the physiological salt solution followed by 4% formaldehyde. Subsequently, brains were removed and used for histological analysis, including sectioning and staining, to confirm the proper positioning of electrodes on the FRM to eliminate unexpected data (Supplementary Figure S1A). All studies were conducted following the Guide of Laboratory Animal Management Ordinance of China, and are approved by the Jiangsu Association for Laboratory Animal Science (Permit Number: 2010012).

### 2.3. Experimental procedures

**Figure 2:**
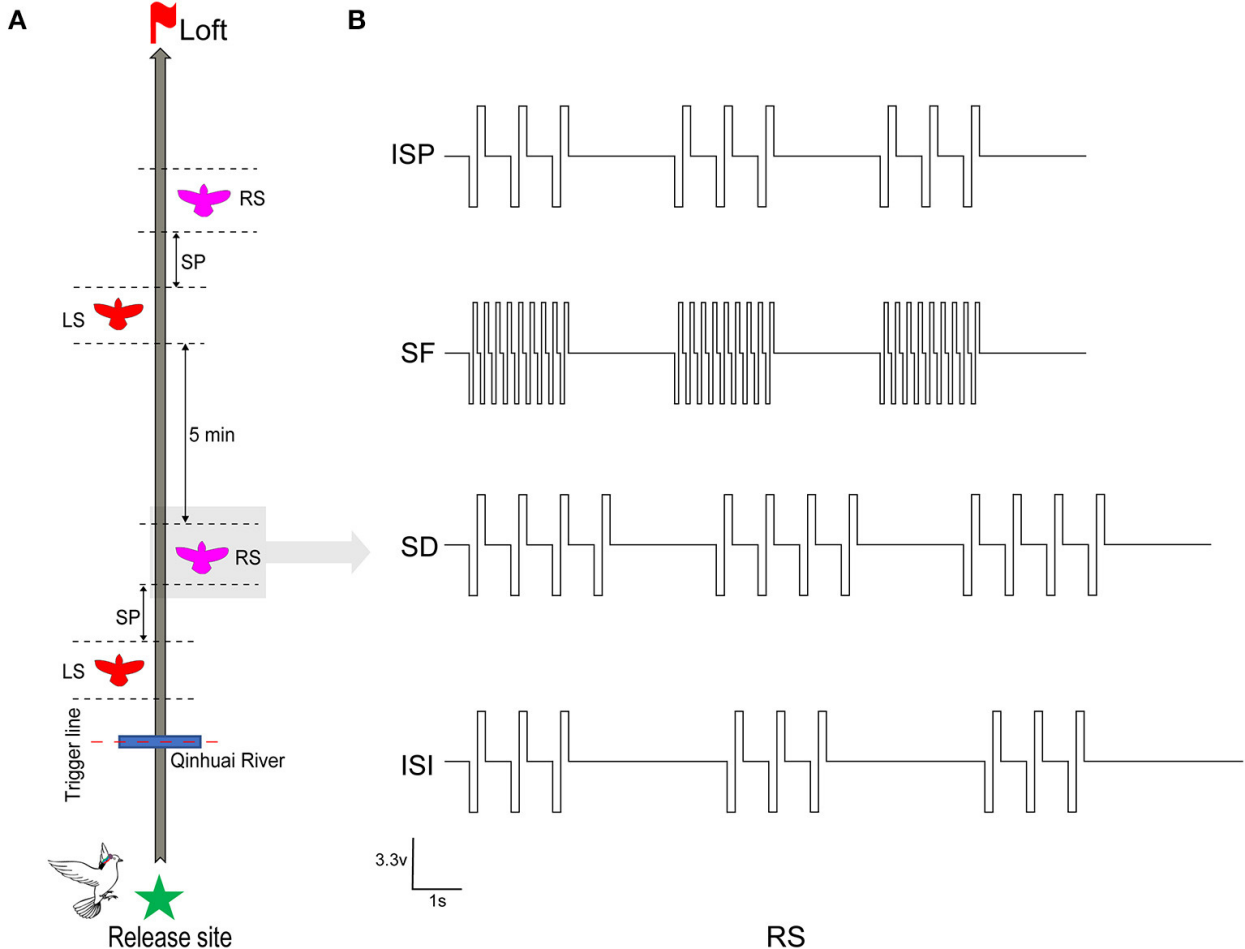
Stimulation procedure and parameters in robo-pigeon. **(A)** The complete stimulus experiment procedures of each robo-pigeon. LS denotes that the robo-pigeon was stimulated in the left-FRM, and RS in the right-FRM. SP denotes the unstimulated silent period between the left stimulus and right stimulus segments. **(B)** The various electrical stimulation parameters used in the present study. ISP, initial stimulation paradigm; SF, stimulus frequency; SD, stimulus duration; ISI, inter-stimulus interval.

Two of the most effective stimuli sites (one in each hemisphere) of all robotic pigeons were selected for steering behavioral control testing to ensure that they were working properly. Between June 2020 and September 2021, on selected sunny days with wind speeds < 2 ms^−1^, all robo-pigeons were surveyed outdoors at 8:00 a.m. The repeatability was assessed to ensure that the drastic changes in the sun rays and weather conditions were not the cause of the observations. Each robo-pigeon will rest for 15 min at the beginning of the experiment and then fly to the loft individually to reduce its free circling flight time in the vicinity of the release site (Taylor et al., 2019). Generally, pigeons start homing after 90–200 s of release, about 200–1,000 m away from the release point. At this time, the flight speed is gradually stable, and the flight path is close to a smooth straight line (Schiffner and Wiltschko, 2009). Afterwards, the robo-pigeons were pre-set to start stimulation experiments when they arrive at Qinhuai River, a landmark on the route, and collect effective flight data (Figure 2A).

The whole stimulus experiment was divided into two sessions, each session contained a left stimulus (LS) and a right stimulus (RS) block respectively, with a 30-s interval between blocks. After the first session, in order to avoid the stimulation fatigue of the animals and ensure that the flight path is straight again, the robo-pigeons were allowed to rest for 5 min before conducting the second session (Figure 2A). Based on our previous experimental experience, the rectangular biphasic square pulse train (cathode leading) was employed for brain stimulation of the robo-pigeons.

### 2.4. Stimulation protocol

To explore the quantitative effects of electrical stimulation parameters on the control of outdoor turning behavior in robo-pigeons, we examined three key parameters: stimulation frequency (SF), stimulation duration (SD), and interstimulus interval (ISI; Figure 2B).

Each parameter consisted of four test levels, as shown in the “Test level” column of Table 1, the SF level is set between 60 and 120 Hz, and the SD and ISI levels are set between 2 and 5 s because robo-pigeons were lost frequently in experiments with SF over 120 Hz or SD over 5 s. The principle of “minimum effective dose” and one-way repeated measures analysis of variance ANOVA were adopted to avoid the influence of individual differences and flight safety. Meanwhile, in order to avoid deviations in electrode implantation sites and individual differences, the SF, SD, and ISI (SF = 60 Hz, SD = 2 s, and ISI = 2 s) were fixed for each individual, and the stimulation pulse width was adjusted so that each subject could make successfully one turn circle indoors during a single stimulation cycle, ensuring a consistent initial turning behavior for each subject.

**Table 1:** Parameter test values.

**Voltage**	**Waveform**	**Test parameter**	**Unit**	**Test levels**
3.3 V	Biphasic square pulse train	Stimulus frequency (SF)	Hz	60, 80, 100, 120
		Stimulus duration (SD)	s	2, 3, 4, 5
		Inter-stimulus interval (ISI)	s	2, 3, 4, 5

Each experiment focused on one parameter to examine the relationship between different stimulation parameters and turning behaviors. The sessional designs were as follows: (1) SF adjusted, the pulse frequency was changed from 60 to 120 Hz with an increasing step size of 20 Hz, and other parameters were fixed at their initial values; (2) SD adjusted, the stimulus duration was changed from 2 to 5 s with an increasing step size of 1 s, while other parameters remain unchanged; (3) ISI adjusted, the inter-stimulus interval was changed from 2 to 5 s with an increasing step size of 1 s, and other parameters were kept constant. Each set of stimulus parameters was tested repetitively at least five times for all subjects. Due to the fact that pseudoreplication might affect the conclusions of statistical analyses in ecological, animal behavior and neuroscience studies, the test order of the respective sessions obeyed a pseudorandomized sequence at the present study (Freeberg and Lucas, 2009; Lazic, 2010).

### 2.5. Data acquisition and processing

The control device of the robo-pigeon was 30 mm × 24 mm × 12 mm (L × W × H), with a total mass of 14.9 g (Supplementary Figure S1B) including a rechargeable lithium battery, which mainly integrates two functions: flight trajectory recording and brain microstimulation. The GPS of the control module device was capable of logging time-stamped longitude, latitude and altitude data at 5 Hz. However, we did not analyze the altitude due to the known data inaccuracy recorded by GPS. Data processing referred to the previous method of Nagy et al. where flight trajectories were converted from the geographic coordinate system to the metric system using Universal Transverse Mercator projection coordinates and then smoothed with a 3-point moving average filter, with occasional missing data points are replenished by average interpolation (Nagy et al., 2010). All data were pre-processed, and those data with no turning response after stimulation or the direction of turning flight is inconsistent with the stimulated brain area were considered as a failure of turning flight control. It was considered that the turning flight control is successful when the location of the brain stimulus was consistent with the direction of the robo-pigeon's turning flight, and the recorded data were validated for further statistical analysis of the turning flight behavior status and calculation of the turning flight control success rate (SR). Meanwhile, the flight data of the non-stimulated segment during the same stimulus time before turning to flight were calculated as a positive control.

### 2.6. Analysis of turn flight behavior

**Figure 3:**
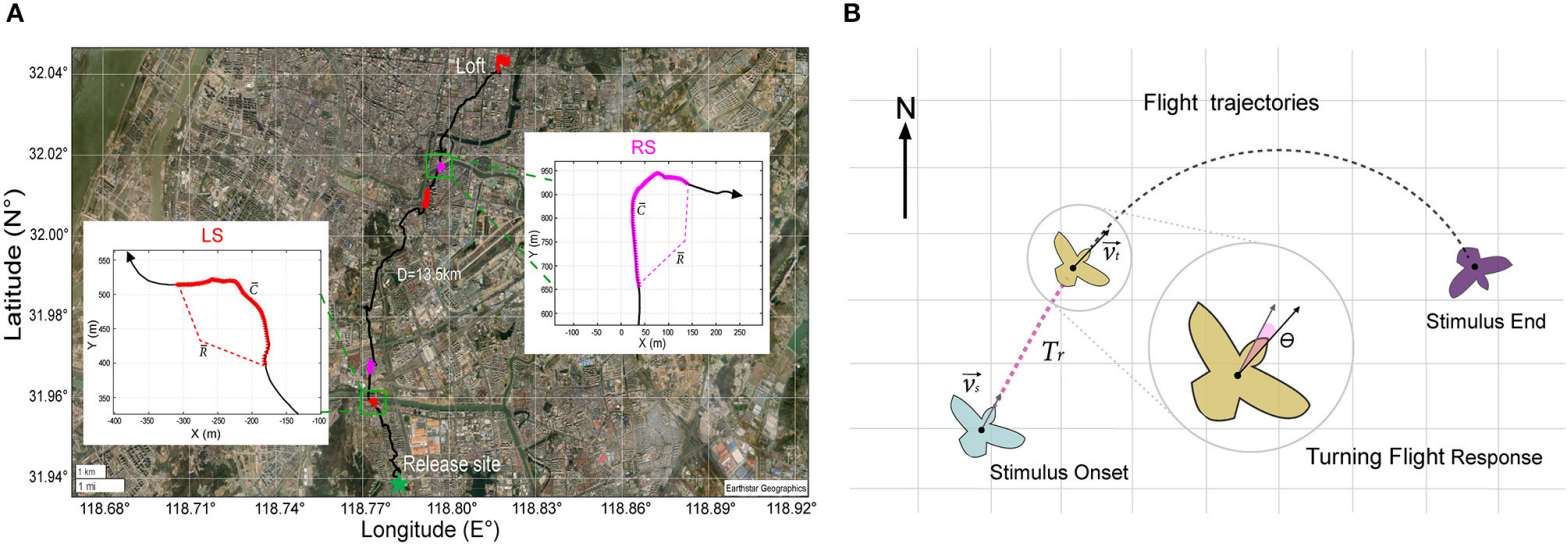
GPS data analysis during turning flight of robo-pigeon. **(A)** Example of stimulated turning flight of a robo-pigeon. The green star denotes the place where the robo-pigeons were released and the red flag was the loft of pigeons. The straight distance between the release site and the loft is over 10 km. Different colors represent different flight trajectories, the black line denotes the flight trajectories of the robo-pigeon without stimulation; the red line denotes the flight trajectories with the stimulation to the left-FRM (LS); the magenta line denotes the flight trajectories with the simulation to the right-FRM (RS). The insets within figures the show the flight trajectory of the robo-pigeon when it was stimulated right and then left FRMs, respectively, under the Universal Transverse Mercator projection coordinate system. $\bar{V}$ denotes the average speed of flight during the phase of stimulation, $\bar{C}$ denotes the average curvature of turning during the phase of stimulation, and $\bar{R}$ denotes the average radius of flight during the phase of stimulation. **(B)** The turning flight variables were calculated from the turning flight trajectories of the robo-pigeon after being stimulated. The light blue, brown, and purple cartoons represent, respectively different turning flight states of the robo-pigeon. Where $\vec{v_{s}}$ denotes the flight speed vector at the stimulus onset and $\vec{\nu_{t}}$ was the flight speed vector at the beginning of the turn. The response time of the robo-pigeon's turning flight behavior was denoted as *T*_*r*_ and the turning angle after being stimulated was denoted by θ.

To provide a quantitative evaluation of the impact of various stimulus parameters on the turning behavior of the robo-pigeon, we computed three key metrics: the average turning flight speed ($\bar{V}$), average turning curvature ($\bar{C}$), and average turning radius ($\bar{R}$) over the entire stimulation period. These metrics were illustrated in Figure 3A. The turning curvature was calculated with the following formula


(1) $$C(t)=|\dot{r}\left(t\right)\times \ddot{r}\left(t\right)|/|\dot{r}\left(t\right){|}^{3}$$

Where ṙ(*t*) and $\ddot{r}\left(t\right)$ corresponded to the first and second derivatives of *r*(*t*), respectively, × was the cross product, and *r*(*t*) denoted the trajectory of the center of mass of the pigeon in the horizontal plane (Nagy et al., 2010). By the above formula, we computed the average turn curvature ($\overset{\bar{}}{C}$) for each pigeon throughout the entire stimulation period. Furthermore, we calculated the reciprocal of $\overset{\bar{}}{C}$ to determine the average turning radius ($\overset{\bar{}}{R}$), expressed as $\overset{\bar{}}{R}=1/\overset{\bar{}}{C}$. This approach allowed us to quantify and analyze the robo-pigeon' turning behavior with precision and accuracy.

To further characterize the turning flight state of the robo-pigeons after stimulation, we introduced two measures: turning behavior response time (*T*_*r*_) and turning angle (θ), as shown in Figure 3B. We defined *T*_*r*_ as the time when the flight direction changed significantly in the turning flight trajectory of the robo-pigeons after stimulation was presented and measured all valid sample data. To calculate the turning angle, we used the formula


(2) $$\theta =acos(\vec{v_{s}}\cdot \vec{\nu_{t}})/\left(\begin{array}{c} \left|\vec{v_{s}}\right|\cdot \left|\vec{\nu_{t}}\right| \end{array}\right)$$

where the $\vec{v_{s}}$ and $\vec{\nu_{t}}$ is the vector dot product (Pettit et al., 2013). These measures provide a quantitative way to assess the turning behavior of the robo-pigeons in response to the presented stimulus. All analyses were conducted on the platform Matlab 2019b (The MathWorks, Inc., Natick, MA, USA).

### 2.7. Statistics analyses

The successful turning flights were counted for each subject, and the data were presented as the mean ± standard error. Of distribution normality of means and homogeneity of variances of flight speed, turning curvature and turning radius were estimated with the Shapiro–Wilk *W*-test and Levene's test, respectively. No significant main effects for the factor “Stimulate direction” (LS and RS) were found for the turning flight behavior of robo-pigeons, suggesting that the results of the present statistical analyses did not be affected by pseudoreplication. In addition, since the turning behavior data did not meet the statistical assumptions, all data sets were pooled regardless of the “Stimulus direction,” and the Wilcoxon signed-rank test was used for statistical analysis to explore the differences between the subjects' turning behavior and stimulus variables. All analyses were conducted using IBM SPSS Statistics 26.0 (Illinois, USA) using *p* < 0.05 as the significance level (Laird and Mosteller, 1990).

## 3. Results

### 3.1. The control success rate of turning flight

**Figure 4:**
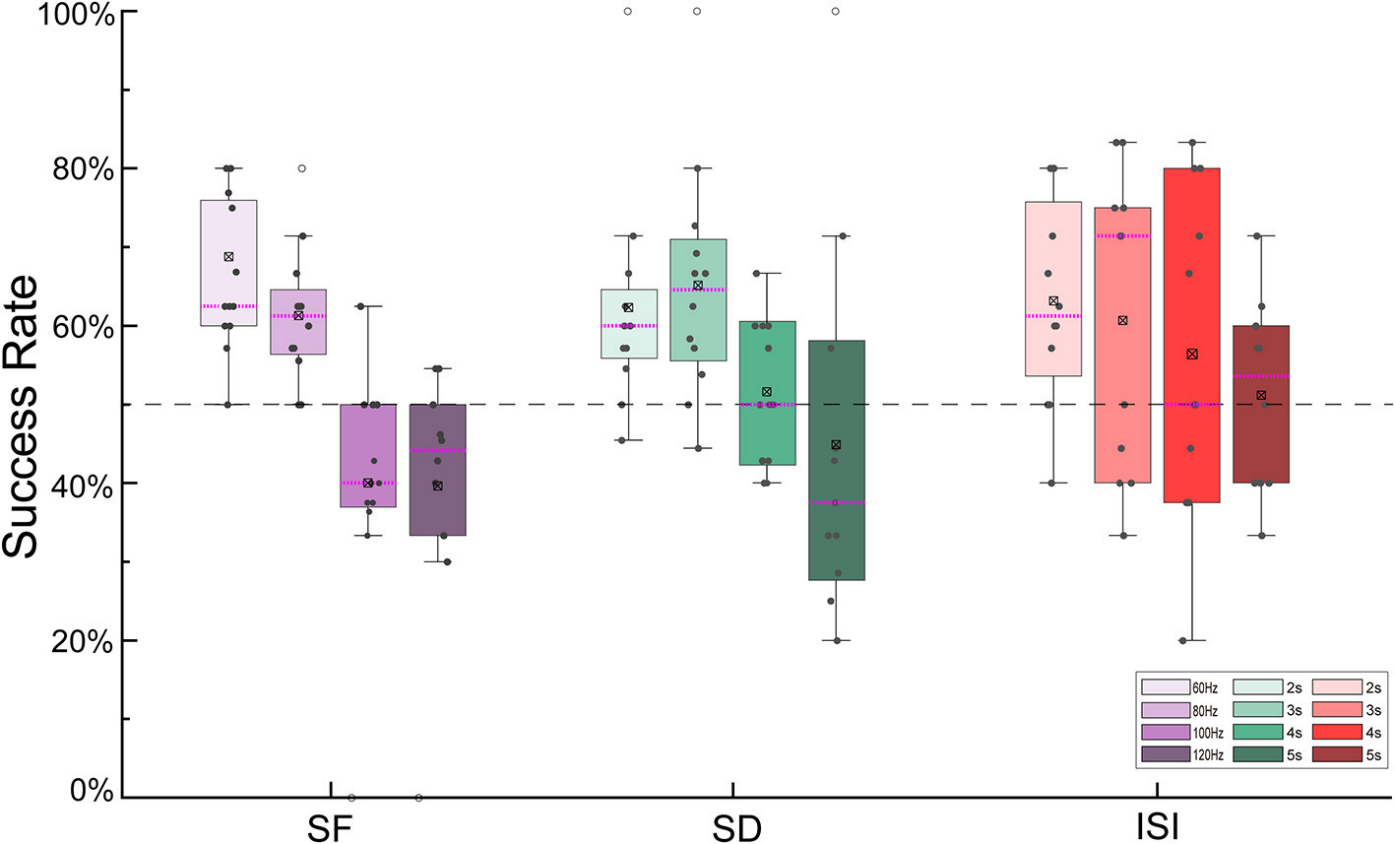
The success rate of turning flights controlled by different stimulus parameters. SF, stimulus frequency; SD, stimulus duration; ISI, inter-stimulus interval.

The success rates of the outdoor turning flight control of the robo-pigeon under different stimulation parameters were shown in Figure 4. For the SF, the maximized success rate (66.10%) of turning flight control was achieved at 60 Hz. In general, with the increasing SF, the success rate of turning flight gradually decreases. While SF exceeding 100 Hz (41.39%) and 120 Hz (39.69%), the success rates of turning flight were significantly reduced (Figure 4, Supplementary Figure S2A and Supplementary Table S1). Similarly for the SD, the success rates of turning flight were over 50% for the stimulation duration of 2–4 s. Among them, the success rates of 62.28 and 65.13% were achieved with 2 and 3 s of SD, respectively, while the success rate was only 44.87% when the SD was 5 s (Figure 4, Supplementary Figure S2B and Supplementary Table S2). For the ISI, the success rates of turning flight were over 50% in all trials. However, the success rate gradually decreases when the inter-stimulus interval exceeded 4 s (Figure 4, Supplementary Figure S2C and Supplementary Table S3).

### 3.2. Effects of varied electrical stimulation parameters

**Figure 5:**
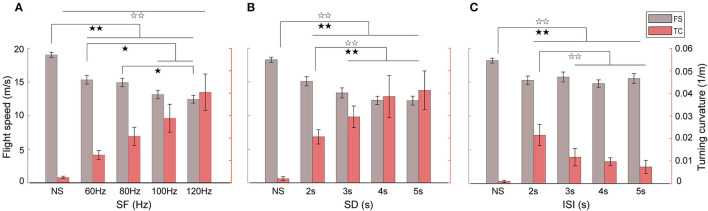
Effects of stimulus parameter variations on the turning speed and curvature of robo-pigeons. **(A)** With different stimulus frequencies; **(B)** with different stimulus duration; **(C)** with different inter-stimulus intervals; NS denotes the non-stimulus segment before turning flight; SF, stimulus frequency; SD, stimulus duration; ISI, interstimulus interval; FS, flight speed; TC, turning curvature. Black stars indicate significant differences in the flight speed, and blank stars significant differences in the turning curvature, respectively (^*^p < 0.05, ^**^p < 0.01).

Different behavioral responses in flight speed and turning curvature of robo-pigeons were attributed correspondingly to stimulation parameters that varied in the present study (Figure 5 and Table 2). The average turning flight speed of the robo-pigeon gradually decreased with the increase of stimulation frequency while keeping the other parameters unchanged. Especially, there was a significant difference in the flight velocity to stimuli between low-frequency (60 and 80 Hz) and high-frequency (100 and 120 Hz; Figure 5A and Supplementary Table S4). Meanwhile, the average turning flight curvature gradually increased along the stimulation frequency, and the turning flight curvatures were significant difference to varied stimulus parameters (Figure 5A and Supplementary Table S4).

**Table 2:** Results of the robo-pigeons turning flight controlled with the varied parameters of SF, SD and ISI.

**Stimulus parameters**	***T_*r*_*(s)**	**θ (deg)**	**$\bar{V}$ (m/s)**	**$\bar{C}$ (m^−1^)**	**$\bar{R}$ (m)**
**SF**					
NS	N/A	N/A	18.584 (±0.377)	0.002 (±0.001)	N/A
60 Hz	4.484 (±0.251)	±23.331 (±1.237)	15.337 (±0.645)	0.012 (±0.002)	80.206 (±5.541)
80 Hz	4.106 (±0.218)	±32.873 (±2.360)	14.935 (±0.630)	0.021 (±0.004)	48.028 (±8.440)
100 Hz	3.772 (±0.265)	±43.263 (±2.888)	13.150 (±0.648)	0.029 (±0.006)	34.627 (±8.223)
120 Hz	3.739 (±0.241)	±51.277 (±4.243)	12.447 (±0.517)	0.040 (±0.008)	24.725 (±6.258)
**SD**					
NS	N/A	N/A	18.296(±0.330)	0.002 (±0.001)	N/A
2 s	4.125 (±0.261)	±33.479 (±2.850)	15.085(±0.561)	0.021 (±0.003)	48.702 (±11.125)
3 s	4.008 (±0.240)	±40.609 (±3.068)	13.367(±0.620)	0.029 (±0.004)	33.927 (±7.008)
4 s	3.822 (±0.313)	±51.167 (±3.249)	12.560(±0.625)	0.039 (±0.009)	25.968 (±8.768)
5 s	3.791 (±0.382)	±51.555 (±4.428)	12.215(±0.683)	0.041 (±0.009)	24.234 (±6.161)
**ISI**					
NS	N//A	N/A	18.182 (±0.359)	0.001 (±0.000)	N/A
2 s	4.151 (±0.196)	±32.915 (±2.148)	15.259 (±0.634)	0.022 (±0.004)	46.490 (±8.035)
3 s	4.357 (±0.324)	±25.360 (±1.557)	15.756 (±0.740)	0.012 (±0.004)	85.317 (±13.918)
4 s	5.325 (±0.353)	±20.996 (±1.209)	14.785 (±0.524)	0.009 (±0.002)	102.634 (±10.293)
5 s	5.543 (±0.377)	±16.998 (±0.857)	15.541 (±0.721)	0.007 (±0.003)	135.471 (±12.800)

SF, the stimulus frequency; SD, the stimulus duration; ISI, the inter-stimulus interval; NS, the non-stimulus segment before turning flight; T_r_, the response time of turning flight behavior; $\bar{V}$, the average flight speed; $\bar{C}$, the average turning curvature turning flight; $\bar{R}$, the average turning radius of turning flight; N/A, not applicable.

An increase in stimulus duration resulted in a decrease in turning flight speed. However, after the stimulus duration was >4 s, the turning flight speed decreased and leveled off, and there was no longer a significant difference (Figure 5B and Supplementary Table S4). Interestingly, the changing trend in the turning flight curvature of the robo-pigeon was correlated reversely with the turning flight speed. With the increase of the stimulus duration, the flight curvature of the robo-pigeon gradually increased, but in conditions of the stimulus durations >4 s, the curvature stabilized progressively (Figure 5B and Supplementary Table S4). When the SD exceeded 5 s, the robo-pigeon could not complete an effective turning flight response (Figure 4).

The turning flight curvature of the robo-pigeon gradually decreased as the inter-stimulus interval increased. However, for the turning flight speed, there were no significant differences in response to the varied inter-stimulus interval (Figure 5C and Supplementary Table S4). With the stimulus duration set to 2 s, the silent period was elongated as the ISI increased eventually. During the silent period of stimulation, the robo-pigeons automatically accelerated their flight, which has no significant effect on the turning flight speed, but the turning curvature has a gradually decreasing trend (Figure 5). These results indicated that SF and SD were key factors in the activation of FRM neurons in the target nucleus and played a major role in the turning flight speed and turning curvature of the robo-pigeon, while the ISI only has a significant effect on the control of turning curvature.

### 3.3. Turning flight behaviors to stimuli with different parameters

**Figure 6:**
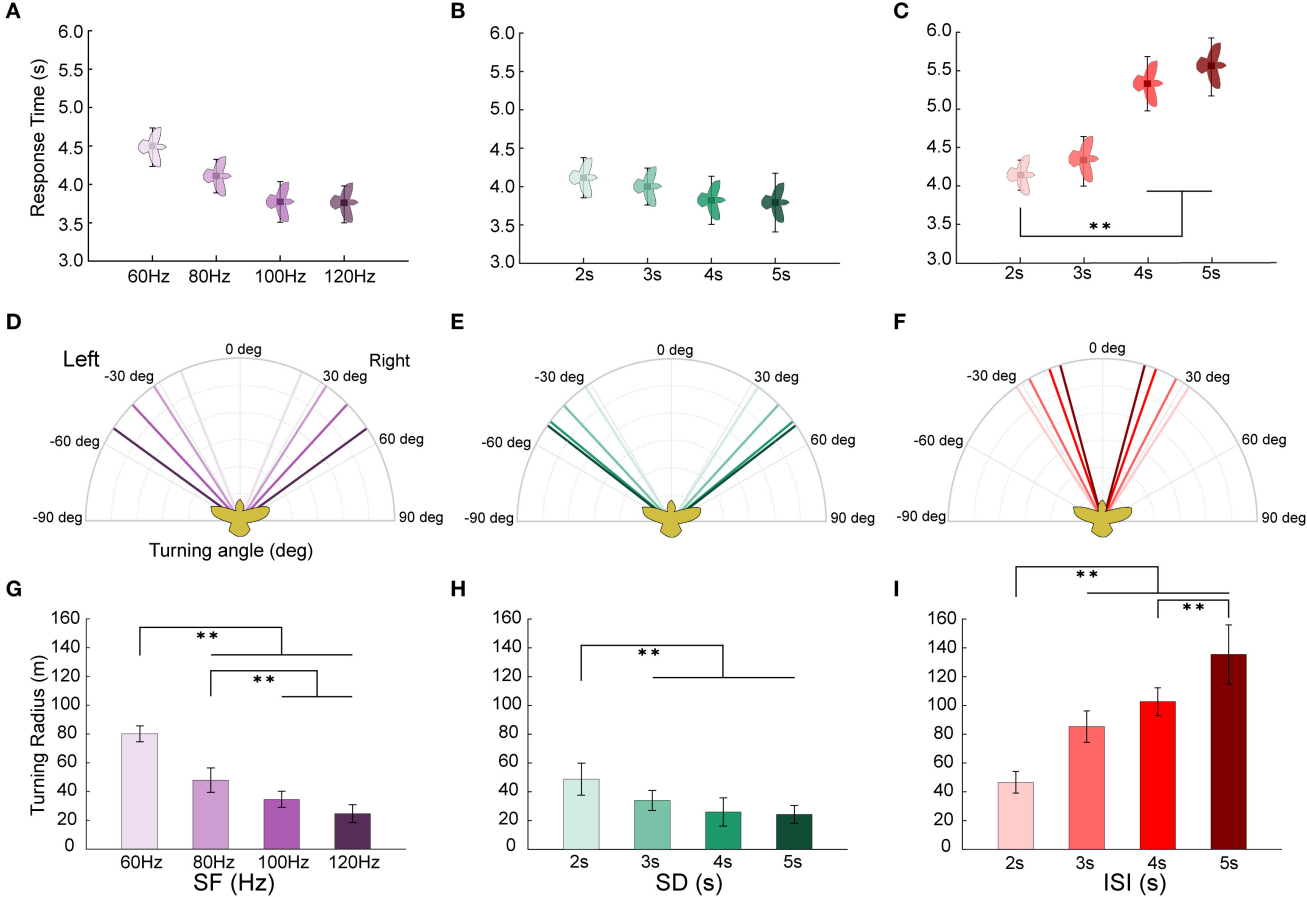
Turning flight states of robo-pigeons after being stimulated. **(A, D, G)** denote the response time, turning angle and turning radius under the stimulus parameters of SF, respectively. **(B, E, H)** denote the response time, turning angle and turning radius under the stimulus parameters of SD, respectively. **(C, F, I)** denote the response time, turning angle and turning radius under the stimulus parameters of ISI, respectively. SF, the stimulus frequency; SD, the stimulus duration; ISI, the inter-stimulus interval. Each asterisk indicate significant differences in response time and turning radius, respectively (^*^p < 0.05, ^**^p < 0.01).

The turning response time, turning angle, and turning radius of the robo-pigeon are likely to vary with different stimulus parameters. Of the reaction time of turning flight, although there was no statistically significant difference among different stimulation frequencies, it trended that the reaction time accelerated as the stimulus frequency increased (Figure 6A, Table 2 and Supplementary Table S4). Similarly, no statistically significant difference in response to the varied stimulation durations, while it tended obviously to decrease with the increase of stimulation duration and reached a plateau when the stimulation exceeded 4 s (Figure 6B, Table 2 and Supplementary Table S4). In addition, increments of the inter-stimulus interval could significantly elongate the reaction time of the robo-pigeons (Figure 6C, Table 2 and Supplementary Table S4).

The turning angle could gradually rise as the stimulus frequency increased. In general, under the appropriate stimulation frequency, the average turning angle could be controlled at 20–55° (Figure 6D and Table 2). The increments of stimulus duration could lead to an increase in the turning angles for which under varied ranges applied in the present study the average turning angles could be adjusted from 30 to 55° (Figure 6E and Table 2). The variation in turning angle became smaller when the inter-stimulus interval was raised and using those ISI variations in our study the average turning angle could be controlled between 15 and 35° (Figure 6F and Table 2).

The turning radius gradually decreased with steadily increasing stimulation frequency (Figure 6G, and Supplementary Table S4) and under varied extents applied in our study, the average turning radius could be adjusted to a range of 25–80 m (Table 2). An increase in the stimulus duration could cause a decrease in the turning radius of the robo-pigeon, while under those stimulations with a duration longer than 4 s the turning radius was gradually fixed, resulting in no significant difference seen in response to different SDs (Figure 6H and Supplementary Table S4). Changing SDs within a range applied in our study the average turning radius could be adjusted from 25 to 50 m (Table 2). The robo-pigeons flew generally in a larger turning radius when receiving the shorter inter-stimulus intervals (Figure 6I and Supplementary Table S4). With varied ISI of the present study, the average turning flight radius could be controlled between 45 and 135 m (Table 2).

### 3.4. Personalized turning flight characteristics

**Figure 7:**
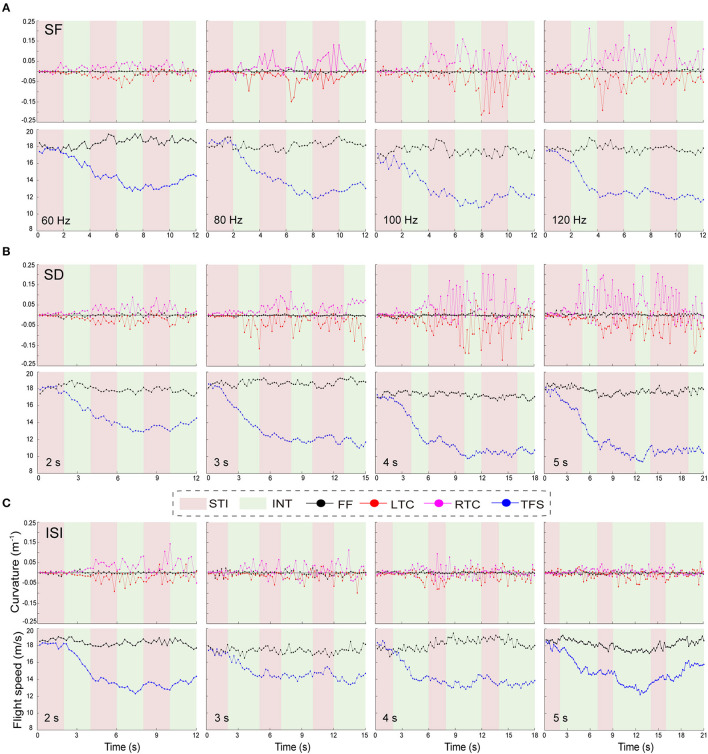
Characteristics of the turning flight behavior of the robo-pigeon during the complete stimulation cycle. **(A–C)** indicate the variation of turning flight curvature and turning flight speed of the robo-pigeon within different parameters of SF, SD and ISI, respectively. SF, the stimulus frequency; SD, the stimulus duration; ISI, the inter-stimulus interval; STI, denote the stimulation (red regions); INT, denote the stimulation interval (green regions); FF, the flight speed and curvature of the robo-pigeon in free flight; LTC; the curvature of left-turning; RTC; the curvature of right-turning; TFS, the speed of turning flight.

The results showed that differences of stimulation parameters directly affected the turning behavior of the individual robo-pigeons, or in other word, the turning behavior of each robo-pigeon has stereotyped and individualized characteristics during the whole stimulation process (Figure 7). After being stimulated, the flying pigeon did not turn immediately, but decelerated first and then turned. Among the three stimulus cycles of the experiment, the first stimulus cycle had significant behavioral variability compared to the two subsequent stimulus cycles.

Specifically, the robo-pigeons usually decelerated rapidly when the first stimulus occurred, and the deceleration rate was strongly positively correlated with the SFs and SDs. Compared with the flight speed of the unstimulated segment, the flight speed during the turned ranged of ~12–15 m/s. The higher the stimulus intensity (SF and SD), the lower the speed when turning. During this stimulation cycle, the turning rate of the robot pigeon did not change significantly, and the flight trajectory basically tended to fly in a straight line (Figure 7). Notably, the turning behavior mainly occurred during the two subsequent stimulus cycles, and the turning flight speed of a robo-pigeon remained stable as the turn occurred. The level of the stimulus parameters did not appear to have a significant deceleration effect on the flight speed of the robo-pigeons. At the same time, we could clearly find that the turning curvature rate of the robo-pigeon had changed significantly, and the greater the stimulus intensity (SF and SD), the higher the dispersion of the curvature change. In contrast, under the ISI, the curvature change became tighter as the stimulus interval increased during turning (Figures 7A, B). Meanwhile, the shorter stimulus interval had no significant effect on the flight speed during the turning process, but when the ISI was set to 5 s, the turning flight speed of the robo-pigeon would significantly accelerate the flight process in the second half (Figure 7C).

## 4. Discussion

### 4.1. General perspectives

Unlike indoor studies, small stimulus changes in the outdoor execution led to significant behavioral changes. In order to ensure the safety, stability, and accurately control of the outdoor steering flight behavior of the robo-pigeons, this study investigated the effects of different electrical stimulation parameters (SF, SD, and ISI) on the steering flight behavior of the robot pigeon. Generally, when controlling the turning behavior of the robo-pigeon indoors, the turning behavior occurred immediately after stimulation onset, and the turning speed and turning angle were significantly correlated with the parameters of SF and SD. The higher the SF and SD were, the faster the turning speed and the more pronounced the turning angle appeared. This is consistent with previous studies on the control of the turning behaviors of robo-rats by electrical stimulation in the VPM region, confirming that the electrical stimulation parameters of SF and SD are the key factors in activating neuronal excitatory activities (Xu et al., 2016). There is likely a significant linear or close linear relationship between the turning angle of rat-robot and stimulation parameters.

Previous electrophysiological studies have shown that the electrical activation of neurons depends on the amount of current flowing directly through the electrode that is proportional to the square of the distance between the neuron and the electrode tip, while the degree of neuron activation is related to the amount of effective charge applied (Fouriezos and Wise, 1984; Yeomans et al., 1986; Tehovnik, 1996). Increasing the intensity of electrical stimulation parameters (SF, SD) can activate a larger volume of neurons, which is the main reason for the significant enhancement of turning behaviors. In general, neuronal nuclei are composed of different functional subregions containing different types (excitatory/inhibitory) of neuronal cells (Ranck Jr, 1975; Overstreet et al., 2013; Tremblay et al., 2016). Electrical stimulation primarily acts on axons and dendrites near electrodes, rather than on somatic cells with higher stimulation thresholds (Tehovnik, 1996).

#### 4.1.1. Stimulus frequency

Different from the static turning control of the robo-rats on the ground, the turning flight control of the robo-pigeons outdoors is in the process of flying in the air. After being stimulated, they will decelerate first and then turn (Figure 7A). When faced with different locomotor environments (ground/air), the animal's motor regulatory system selectively activates the different functional subregions of the target nuclei as well as specific motor neural pathways to adapt to the demands of turning in different environments. Therefore, while in the air, with increasing SF parameters, the pigeons' turning curvature gradually increases, while the turning speed decreases significantly. The deceleration ratio of the flight speed is closely related to the SF parameter. As the SFs increase, the turning flight speed of the robo-pigeons will decrease, due to the fact that the nervous system does not allow arbitrary increases in response to stimulation frequency, e.g., each specific neuronal tissue has its specific excitation activation frequency (Perge et al., 2012; Jamali et al., 2019).

In general, neurons with broad functional and structural properties (i.e., complex tasks, diverse types, and complex physical shapes) have wider frequency bandwidths, while motor neurons responsible for simple and specific tasks have narrower frequency bandwidths of activation (Perge et al., 2012; Jamali et al., 2019). For example, in directional motor control in carp (*Cyprinus carpio*) and cockroach (*Gromphadorhina portentosa*), the nervous system responds specifically between 30–50 and 40–105 Hz, respectively (Zhao et al., 2022). Animal studies have shown that lower stimulation frequencies do not activate neuronal excitability, while mild stimulation within the stimulation frequency ranges of 15–50 Hz for cats, 50 Hz for rats, and 20–50 Hz for pigs can activate the neurons of MLR for motor-behavioral responses (Bachmann et al., 2013; Opris et al., 2019; Chang et al., 2021). High-frequency stimulation can cause persistent depolarization of neural membranes, which inactivates sodium channels, and increase potassium currents to prevent the initiation or propagation of action potentials (i.e., depolarization block), causing neuronal fatigue effects and inability to complete behavioral reactions (Dostrovsky and Lozano, 2002; Magarinos-Ascone et al., 2002; Shin et al., 2007; Bari et al., 2013).

Our results were supported by the previous studies on microstimulation parameters and forelimb motor evoked potentials (MEP) in the rat motor cortex. It has been shown that there was no significant difference in motor thresholds when the stimulus frequency was presented at 142–400 Hz (Young et al., 2011), but no movement is found when the stimulation frequency is below 125 Hz or above 500 Hz (Watson et al., 2016b). Furthermore, due to individual differences, the high stimulation frequency will cause the robo-pigeon to lose the centripetal acceleration required for turning in a short time, sometimes causing the pigeons to fully stop flying (Ros et al., 2011; Read et al., 2016). Therefore, combined with our results, the up-frequency limit of the turning flight control stimulus of the robo-pigeon appears at 100 Hz, above which the turning success rate will drop significantly (Figure 4).

#### 4.1.2. Stimulus duration

With the increase of SD, the turning flight speed of the pigeon gradually decreased, and the average turning flight speed reached a plateau when the SD exceeded 4 s (Figure 5B). In addition, there was no significant difference in the average turning curvature at this condition. The underlying mechanism of electrical stimulation may be attributed to the depolarization process of neuronal membranes. Electrical stimulation through the cathode causes the flow of positive intracellular charge to the outside of the cell, lowering the membrane potential, and leading to depolarization and thus tissue excitation (Adrian, 1968). Previous experiments have shown that for a stimulus being able to cause excitation in tissue cells, the minimum stimulus intensity for generation of an action potential (i.e., the threshold) must be reached. For a short period of time, cells are excited after receiving a single stimulus, and their excitability will undergo a series of changes before returning to normal (Schmidt et al., 1996; Bartlett et al., 2005).

Thus, with increasing SDs, sustained charge accumulation leads to the refractory period of stimulated nuclei (Atrens and Cobbin, 1976). That is, the excitability threshold will be infinitely high during refractory period, especially during absolute undershoot, often requiring the use of stimulation intensities over controls to re-induce tissue excitation (Adrian, 1968; Tehovnik, 1996; Watson et al., 2016b). In general, the number of directly activated neurons increases with SD, probably due to prolonged stimulation generating large currents that spread through multi-synaptic projections in the specific brain areas (Watson et al., 2016a) that innervate extra muscle groups whose coordinated activation may produce complex movements (Gioanni and Lamarche, 1985; Neafsey et al., 1986), which in turn indirectly affected the turning flight control of the robo-pigeon.

#### 4.1.3. Inter-stimulus interval

In this study, when the defaulted SD was set to 2 s, the ISI parameter was set to 2–4 s, which has sufficient stability to facilitate the turning flight control of the robo-pigeon (Figure 4). In neuronal cells, the phospholipid bilayer on the cell membrane is a unique structure that gives neurons a certain capacitance (Nagle and Tristram-Nagle, 2000).

Neurons undergo a time course similar to charge and discharge in response to electrical stimulation. When stimulation occurs the excitability of neuronal cells is activated to generate action potentials after reaching a threshold, and when stimulation is terminated the excitability of the neuron gradually returns to normal into a resting state. For example, in a previous study on the directional motion control of cockroaches, it was found that most of the motor responses occurred within the first 3–4 s after stimulus onset, and when the stimulus was terminated the motor responses gradually diminished within 1.5–2.5 s (Erickson et al., 2015). In the present study, SD of 2 s as the default setting could make the neurons excitation which was sustained for a certain period. The robo-pigeons could complete a turning flight behavior attributing to a prolonged period of neuronal excitation which stepped over the short ISI under condition of the higher stimulus effect. However, when the ISI was large (ISI > 5), the stimulus-activated neuron could step into the resting state for recovering its excitability after the stimulation effect was over. After the stimulus effect wore on, the robo-pigeon actively accelerated its flight and then, the stimulus effect was unable to uphold a turning flight behavior even if the stimulus started once more.

#### 4.1.4. Specific turning flight behaviors

The turning flight behaviors of robo-pigeons in the air showed personalized behavioral characteristics. After being stimulated, it first decelerated before turning. This is because when the pigeon is moving at high speed in the air, it takes a certain amount of time to adjust its flight posture from the point of receiving the turning flight command to the actual change of the flight trajectory, so as to meet the aerodynamic force and turning centripetal force required for turning flight (Ros et al., 2015). Meanwhile, the latency period exists between the start of sensory nerve stimulation and the execution of the motor behavior. The latency of the MEP response in rats decreased when stimulation frequency and pulse duration increased, which occurred exclusively within the effective stimulation ranges while for those stimuli with stimulation frequency >200 Hz or pulse duration >0.34 ms, no such a change could be found (Watson et al., 2016a,b).

During the entire turning flight process, the pigeon needs to coordinate the generation of aerodynamic force and the change of body direction to meet the needs of the turning flight (Hedrick and Biewener, 2007; Ros et al., 2015; Ros and Biewener, 2017; Taylor et al., 2019). The change of body direction is mainly to change the direction of aerodynamic force generation, thereby changing its flight trajectory (Ros and Biewener, 2017). Simultaneously, using the asymmetric wing-beat motion generates enough aerodynamic force to counteract gravity to maintain the turning flight speed. In the following two stimulation cycles, when the pigeons performed the turning flight, the turning curvature rate changed continuously with the adjustment of the body direction, while the turning flight speed tended to be stable. This is actually to ensure the stability of its centripetal acceleration and yaw moment during the turning process, thereby improving the turning performance.

### 4.2. Grade-controlling turning flights

In general, based on the turning behavior characteristics of robo-pigeons, the optimal stimulation parameters to control the turning flight behavior outdoors should be, in a single cycle, less 100 Hz for SF and 5 s for SD, respectively. From the perspective of practical application, to control the robot pigeon to complete the detection and rescue operation of the predetermined target, it is necessary to accurately quantify the turning flight behavior of the robo-pigeons. This requires not only knowing how the turning angle of the robo-pigeons after being stimulated with varied stimulus parameters, but also knowing the relationships between the turning radius and the stimulus parameters. The use of electrical stimulation to control the motion behavior of animal robots has the advantages of fast speed, easy implementation, high repeatability, and sensitive response. It has been shown that the robo-pigeons have highly stereotyped response characteristics to each electrical stimulus parameter (Figure 6). This is consistent with previous studies of turning control in cockroach robots and robo-rats, which made sharper turns at higher electrical stimulation intensities and larger turns at lower electrical stimulation intensities (Erickson et al., 2015; Xu et al., 2016).

Accordingly, combined with the quantitative relationships between the input and output of stimulation parameters (SF, SD, and ISI) and the success rates of turning flight control, the optimal stimulation parameters can be selected to control the turning angle and turning radius of the robo-pigeon. For example, by adjusting the stimulus parameters ISI and SF, the turning radius of the robot pigeon can be regulated in a graded manner over a range of 25–30 m, 45–50 m, 80–85 m, and 130–135 m, respectively (Table 2). Varying the level of the stimulus parameters SF and SD, the turning angle of the robot pigeon will be regulated in a graded manner from 15–25°, 25–35°, 35–45°, and 45–55°, respectively (Table 2). The potential application of the present results is that the optimal electrical stimulation parameters can be selected to control the turning angle and turning radius of the robo-pigeons hierarchically according to the orientation and distance of the target locations. This can not only improve the success rate of the turning flights of robo-pigeons but also reduce the power consumption of the stimulator and the physiological damage to the robo-pigeons caused by electrical stimulation.

## 5. Conclusions

In conclusion, this study quantifies the roles of each stimulus parameter in the graded control of the turning flight behaviors and provides a useful reference for precise turning flight control of the robo-pigeons. The SF and SD parameters have a significant impact on the control of the turning angle of the robo-pigeons, and the change of the ISI parameter can effectively control their turning radius. By quantifying the characteristic relationships between the input stimulus parameters and the output behavior, the turning angle and turning radius of the pigeon can be controlled hierarchically. The current findings can help to realize potentially the practical application of robo-pigeons. Our future work will further quantify the input-output relationship between different stimulus parameter combinations and turning flight radius and provide a parameter interaction model to optimize the stimulus strategy of the robo-pigeon.

## Data availability statement

The original contributions presented in the study are included in the article/Supplementary material, further inquiries can be directed to the corresponding authors.

## Ethics statement

All studies were conducted following the Guide of Laboratory Animal Management Ordinance of China and are approved by the Jiangsu Association for Laboratory Animal Science (Permit Number: 2010012).

## Author contributions

KF, HW, and ZD conceived the study. KF, HW, ZD, and ZW designed the research. HW provides equipment for data acquisition. KF performed the animal surgery, data collection, and drafted the manuscript. KF and ZW carried out data analysis. YT, HM, ZW, WW, and ZD revised the manuscript. All authors read and approved the final manuscript.
