# Supplementary material for: Grade-control outdoor turning flight of robo-pigeon with quantitative stimulus parameters
Source: Front Neurorobot. 2023 Apr 17;17:1143601. doi: 10.3389/fnbot.2023.1143601 (PMC10149694; doi:10.3389/fnbot.2023.1143601)
Supplement: Supplementary file 1 [file Presentation_1.pdf]

Supplementary Material

Grade-Control Outdoor Turning Flight of Robo-pigeon with Quantitative Stimulus Parameters

Ke Fang[1], Hao Mei[1], Yezhong Tang[1, 2], Wenbo Wang[1], Hao Wang[1], Zhouyi Wang[1*], and Zhendong Dai[1]*

*** Correspondence:**
haowang@nuaa.edu.cn
wzyxml@nuaa.edu.cn
zddai@nuaa.edu.cn

1 Supplementary Data

TABLE S1. Number of successful turning flight control trials and success rate for all subjects under parameters of SF.

Subjects	60Hz				80Hz				100Hz				120Hz			
	Trials	Successful	Failed	SR	Trials	Successful	Failed	SR	Trials	Successful	Failed	SR	Trials	Successful	Failed	SR
P_01	8	6	2	75.00%	6	4	2	66.67%	6	3	3	50.00%	5	2	3	40.00%
P_02	10	5	5	50.00%	7	4	3	57.14%	8	4	4	50.00%	13	6	7	46.15%
P_03	5	4	1	80.00%	5	3	2	60.00%	8	5	3	62.50%	11	6	5	54.55%
P_04	7	4	3	57.14%	8	5	3	62.50%	6	2	4	33.33%	10	3	7	30.00%
P_05	8	5	3	62.50%	7	4	3	57.14%	8	3	5	37.50%	11	5	6	45.45%
P_06	5	3	2	60.00%	8	4	4	50.00%	7	3	4	42.86%	11	6	5	54.55%
P_07	13	10	3	76.92%	10	8	2	80.00%	2	0	2	0.00%	3	0	3	0.00%
P_08	8	5	3	62.50%	9	5	4	55.56%	11	4	7	36.36%	12	6	6	50.00%
P_09	12	8	4	66.67%	8	5	3	62.50%	8	3	5	37.50%	9	3	6	33.33%
P_10	10	6	4	60.00%	8	5	3	62.50%	5	2	3	40.00%	N/A	N/A	N/A	N/A
P_11	8	5	3	62.50%	8	4	4	50.00%	5	2	3	40.00%	7	3	4	42.86%
P_12	5	4	1	80.00%	7	5	2	71.43%	4	2	2	50.00%	N/A	N/A	N/A	N/A
**Total**	99	65	34	N/A	91	56	35	N/A	78	33	45	40.00%	92	40	52	39.69%
**Mean±SD**	66.10%±2.58%				61.29%±2.28%				41.39%±4.44%				39.69%±4.21%			

Note: Abbreviations: SF, the stimulus frequency; SR, the success rate of robo-pigeons turning flight control; N/A not applicable.

TABLE S2. Number of successful turning flight control trials and success rate for all subjects under parameters of SD.

Subjects	2s				3s				4s				5s			
	Trials	Successful	Failed	SR	Trials	Successful	Failed	SR	Trials	Successful	Failed	SR	Trials	Successful	Failed	SR
P_01	3	3	0	100.00%	2	2	0	100.00%	5	2	3	40.00%	N/A	N/A	N/A	N/A
P_02	6	4	2	66.67%	13	7	6	53.85%	7	3	4	42.86%	2	2	0	100.00%
P_03	10	6	4	60.00%	8	5	3	62.50%	5	3	2	60.00%	7	2	5	28.57%
P_04	8	5	3	62.50%	6	3	3	50.00%	6	3	3	50.00%	6	2	4	33.33%
P_05	7	4	3	57.14%	9	6	3	66.67%	6	3	3	50.00%	7	4	3	57.14%
P_06	11	6	5	54.55%	5	4	1	80.00%	3	2	1	66.67%	9	4	5	44.44%
P_07	10	5	5	50.00%	12	7	5	58.33%	7	3	4	42.86%	8	2	6	25.00%
P_08	8	5	3	62.50%	11	8	3	72.73%	7	4	3	57.14%	7	5	2	71.43%
P_09	11	5	6	45.45%	9	4	5	44.44%	8	4	4	50.00%	5	1	4	20.00%
P_10	7	5	2	71.43%	9	6	3	66.67%	5	3	2	60.00%	7	3	4	42.86%
P_11	14	8	6	57.14%	7	4	3	57.14%	5	2	3	40.00%	8	3	5	37.50%
P_12	15	9	6	60.00%	13	9	4	69.23%	5	3	2	60.00%	6	2	4	33.33%
**Total**	110	65	45	N/A	104	65	39	N/A	69	35	34	N/A	72	30	42	44.87%
**Mean±SD**	62.28%±4.21%				65.13%±3.94%				51.63%±2.40%				44.87%±6.18%			

Note: Abbreviations: SF, the stimulus duration; SR, the success rate of robo-pigeons turning flight control; N/A not applicable.

TABLE S3. Number of successful turning flight control trials and success rate for all subjects under parameters of ISI.

Subjects	2s				3s				4s				5s			
	Trials	Successful	Failed	SR	Trials	Successful	Failed	SR	Trials	Successful	Failed	SR	Trials	Successful	Failed	SR
P_01	5	3	2	60.00%	5	2	3	40.00%	8	3	5	37.50%	7	5	2	71.43%
P_02	5	4	1	80.00%	8	6	2	75.00%	7	5	2	71.43%	8	5	3	62.50%
P_03	5	2	3	40.00%	5	2	3	40.00%	5	1	4	20.00%	7	4	3	57.14%
P_04	4	2	2	50.00%	6	2	4	33.33%	8	3	5	37.50%	5	2	3	40.00%
P_05	7	5	2	71.43%	6	5	1	83.33%	5	4	1	80.00%	N/A	N/A	N/A	N/A
P_06	9	6	3	66.67%	7	5	2	71.43%	6	3	3	50.00%	5	2	3	40.00%
P_07	7	4	3	57.14%	6	3	3	50.00%	6	3	3	50.00%	6	2	4	33.33%
P_08	6	3	3	50.00%	9	4	5	44.44%	9	4	5	44.44%	5	2	3	40.00%
P_09	8	5	3	62.50%	7	5	2	71.43%	6	4	2	66.67%	6	3	3	50.00%
P_10	5	4	1	80.00%	6	5	1	83.33%	5	4	1	80.00%	5	3	2	60.00%
P_11	5	3	2	60.00%	8	6	2	75.00%	6	5	1	83.33%	7	4	3	57.14%
P_12	5	4	2	80.00%	N/A	N/A	N/A	N/A	N/A	N/A	N/A	N/A	N/A	N/A	N/A	N/A
**Total**	71	45	27	N/A	73	45	28	N/A	71	39	32	N/A	61	32	29	N/A
**Mean±SD**	63.14%±3.46%				60.66%±5.03%				56.44%±5.55%				51.15%±5.15%			

Note: Abbreviations: SF, the inter-stimulus interval; SR, the success rate of robo-pigeons turning flight control; N/A not applicable.

TABLE S4. Results of Wilcoxon signed-rank test for stimulus parameters of SF, SD and ISI.

Parameters	$\bar{V}$				$\bar{C}$				T_r				θ				$\bar{R}$			
**SF**	**60Hz**	**80Hz**	**100Hz**	**120Hz**	**60Hz**	**80Hz**	**100Hz**	**120Hz**	**60Hz**	**80Hz**	**100Hz**	**120Hz**	**60Hz**	**80Hz**	**100Hz**	**120Hz**	**60Hz**	**80Hz**	**100Hz**	**120Hz**
**60Hz**		-0.343	-2.278	-2.769		-3.067	-3.261	-4.301		1.055	-1.341	-1.511		-2.989	-4.319	-4.537		-1.996	-2.757	-3.855
**80Hz**	0.732		-1.689	-2.930	0.002**		-2.135	-3.441	0.291		-0.684	-0.382	0.003**			-3.603	0.046*		-2.260	-3.266
**100Hz**	0.023*	0.091		-0.009	0.001**	0.033*		-2.457	0.180	0.494		-0.178	0.000**	0.001**		-1.496	0.006*	0.024*		-1.275
**120Hz**	0.006**	0.003**	0.993		0.000**	0.001**	0.014*		0.131	0.702	0.859		0.000**	0.000**	0.135		0.000**	0.001*	0.202	
**N**	65	56	33	40	65	56	33	40	65	56	33	40	65	56	33	40	65	56	33	40
**SD**	**2s**	**3s**	**4s**	**5s**	**2s**	**3s**	**4s**	**5s**	**2s**	**3s**	**4s**	**5s**	**2s**	**3s**	**4s**	**5s**	**2s**	**3s**	**4s**	**5s**
**2s**		-2.140	-3.309	-2.303		-2.179	-2.522	-2.396		-0.432	-0.429	-0.487		-1.895	-3.518	-3.195		-2.476	-2.135	-2.411
**3s**	0.032*		-0.983	-1.070	0.029*		-0.803	-0.072	0.666		-0.377	-0.037	0.058		-1.867	-2.475	0.013*		-0.031	-1.359
**4s**	0.001**	0.326		-0.205	0.012*	0.422		-0.381	0.668	0.706		-0.322	0.000**	0.062		-0.521	0.033*	0.975		-0.501
**5s**	0.021*	0.284	0.837		0.017*	0.943	0.704		0.626	0.970	0.747		0.001**	0.013*	0.603		0.016*	0.174	0.616	
**N**	65	65	35	30	65	65	35	30	65	65	35	30	65	65	35	30	65	65	35	30
**ISI**	**2s**	**3s**	**4s**	**5s**	**2s**	**3s**	**4s**	**5s**	**2s**	**3s**	**4s**	**5s**	**2s**	**3s**	**4s**	**5s**	**2s**	**3s**	**4s**	**5s**
**2s**		-0.672	-0.851	-0.206		-2.534	-2.540	-2.263		-0.438	-2.137	-2.185		-2.676	-4.684	-4.463		-2.906	-2.027	-2.900
**3s**	0.502		-0.809	-0.337	0.011*		-0.461	-1.047	0.662		-1.440	-1.651	0.007**		-2.146	-3.347	0.004**		-0.220	-1.842
**4s**	0.395	0.418		-1.402	0.011*	0.645		-1.440	0.033*	0.150		-0.318	0.000**	0.032*		-1.822	0.043*	0.826		-2.670
**5s**	0.837	0.736	0.161		0.024*	0.295	0.150		0.029*	0.099	0.751		0.000**	0.001**	0.068		0.004**	0.065	0.008**	
**N**	45	45	39	32	45	45	39	32	45	45	39	32	45	45	39	32	45	45	39	32

Note: The bottom left indicates the significant difference between the variables, * $p < 0.05$, ** $p < 0.01$; The top right indicates the Rank between the variables. Abbreviations: SF, the stimulus frequency; SD, the stimulus duration; ISI, the inter-stimulus interval; N was the number of trials; $\bar{V}$, the mean flight speed of robo-pigeons; $\bar{C}$, the mean turning curvature of the robo-pigeons turning flight; T_r, the response time of turning flight behavior of robo-pigeons; θ, the mean turning angle of the robo-pigeons turning flight; $\bar{R}$, the mean turning radius of the robo-pigeons turning flight.

2 Supplementary Figures and Tables

2.1 Supplementary Figures

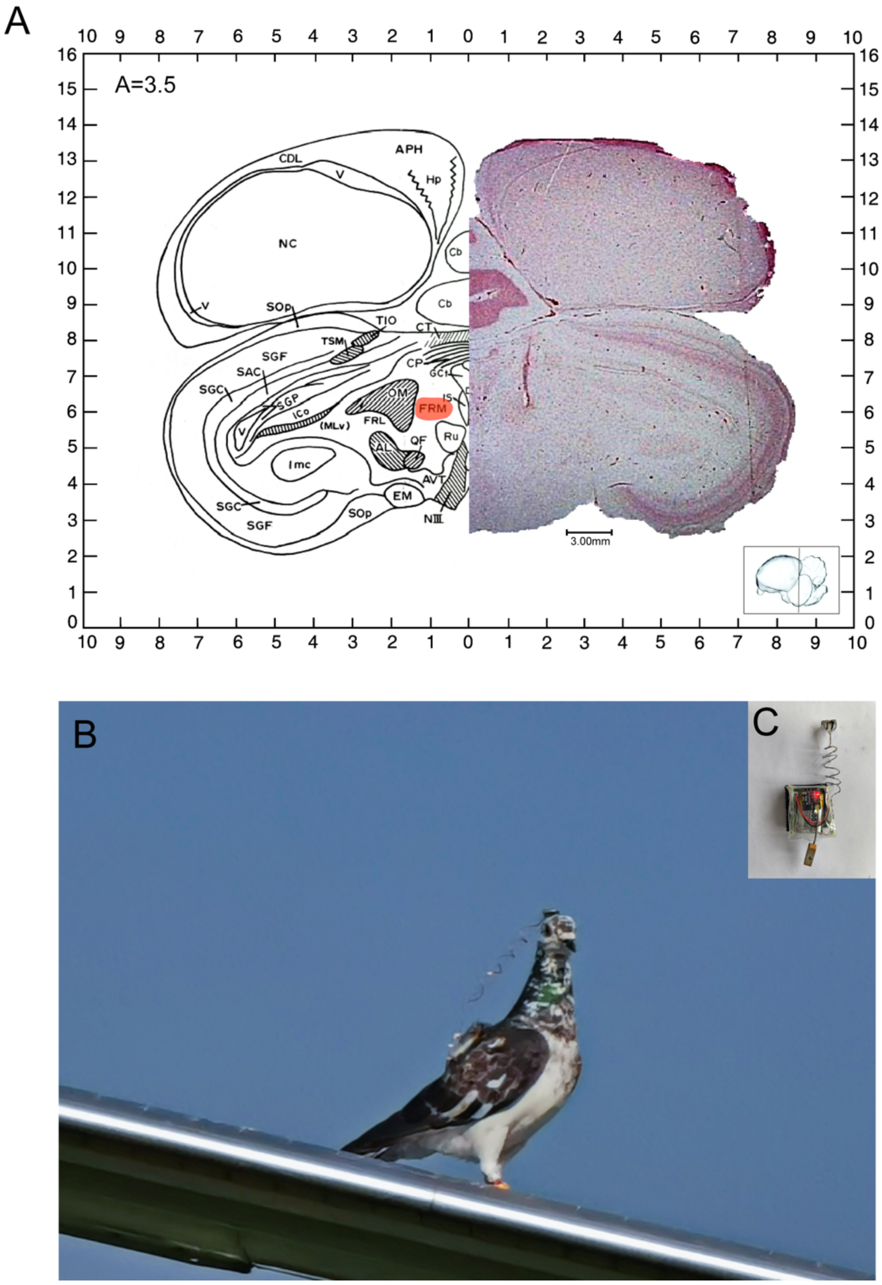

FIGURE. S1 (A) Coronal slices of the pigeon brain demonstrating that the electrode was implanted in the FRM nucleus. (B) A robo-pigeon carrying a control module on its back with a wire connection to the

BCI on the head; (C) The top view of the control module. It is in size of 30 mm × 24mm × 12 mm (L × W × H) and in mass of 14.9 g including a rechargeable battery.

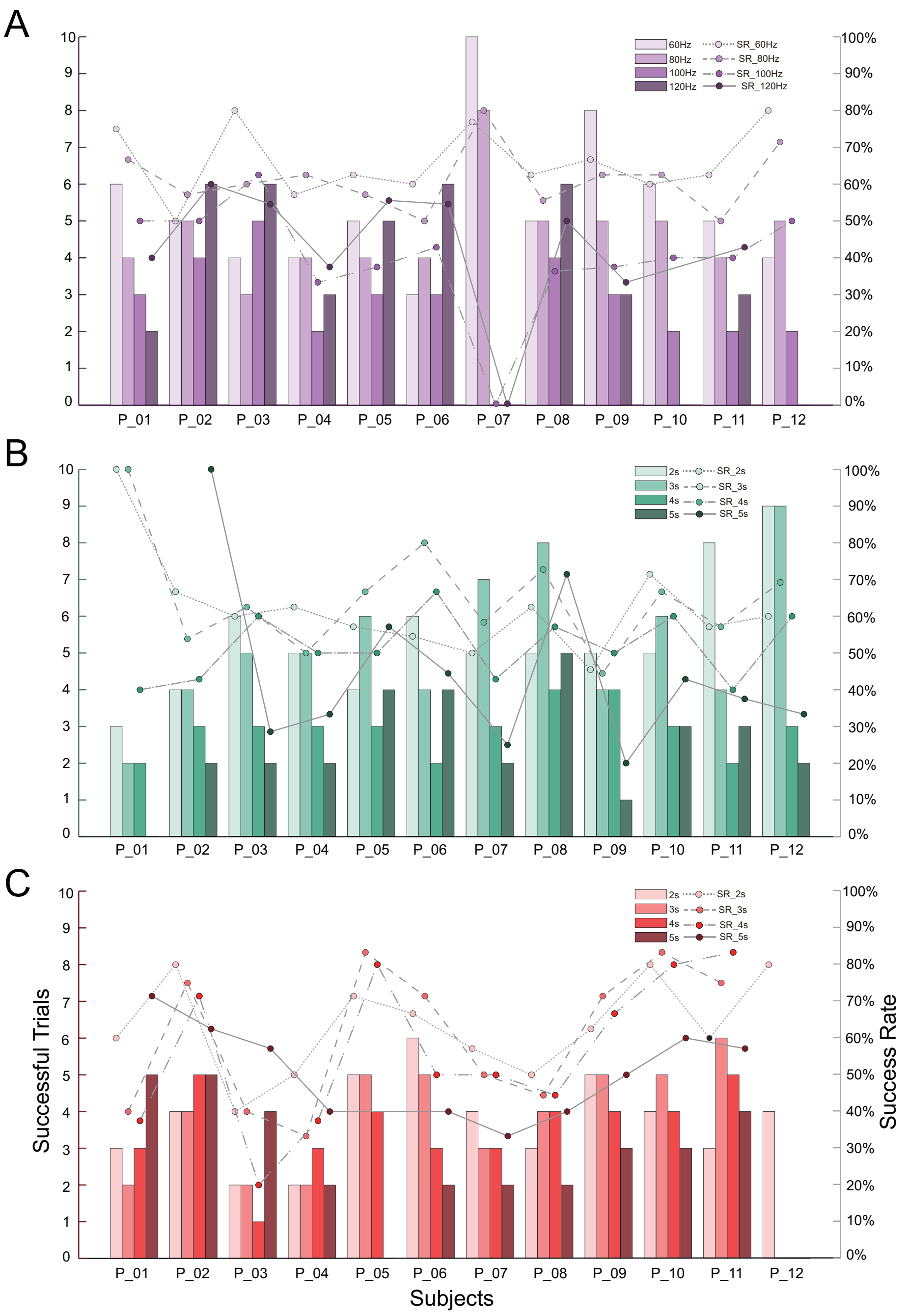

FIGURE. S2 Number of successful turning flight control trials and success rate for all subjects under different stimulation parameters of SF (A), SD (B) and ISI (C).
